# Effects of Neutrophil-to-Lymphocyte Ratio Combined With Interleukin-6 in Predicting 28-Day Mortality in Patients With Sepsis

**DOI:** 10.3389/fimmu.2021.639735

**Published:** 2021-03-16

**Authors:** Shuangqing Liu, Xinkun Wang, Fei She, Wei Zhang, Hongsheng Liu, Xiaodong Zhao

**Affiliations:** ^1^ Department of Emergency, The Fourth Medical Center of the Chinese PLA General Hospital, Beijing, China; ^2^ Department of Radiology, The Fourth Medical Center of the Chinese PLA General Hospital, Beijing, China

**Keywords:** neutrophil-to-lymphocyte ratio, interleukin-6, procalcitonin, mortality, sepsis

## Abstract

**Background:**

The current study aimed to evaluate the relationship between the neutrophil-to-lymphocyte ratio (NLR) combined with interleukin (IL)-6 on admission day and the 28-day mortality of septic patients.

**Material and Methods:**

We conducted an observational retrospective study. Patients with presumed sepsis were included. We observed the correlation of studied biomarkers (NLR, IL-6, PCT, and CRP) and the severity scores (APACHE II and SOFA scores) by plotting scatter plots. The relationships of the studied biomarkers and 28-day mortality were evaluated by using Cox regression model, receiver-operating characteristic (ROC) curve, and reclassification analysis.

**Results:**

A total of 264 patients diagnosed with sepsis were enrolled. It was revealed that IL-6 had the strongest correlation with both APACHE II and SOFA scores, followed by the NLR and PCT, and there was no obvious correlation between CRP and the illness severity. NLR and IL-6 were independent predictors of the 28-day mortality in septic patients in the Cox regression model [NLR, odds ratio 1.281 (95% CI 1.159–1.414), *P* < 0.001; IL-6, odds ratio 1.017 (95% CI 1.005–1.028), *P*=0.004]. The area under the ROC curve (AUC) of NLR, IL-6 and NLR plus IL-6 (NLR_IL-6) was 0.776, 0.849, and 0.904, respectively.

**Conclusion:**

Our study showed that the levels of NLR and IL-6 were significantly higher in the deceased patients with sepsis. NLR and IL-6 appeared to be independent predictors of 28-day mortality in septic patients. Moreover, NLR combined with IL-6 could dramatically enhance the prediction value of 28-day mortality.

## Introduction

Sepsis is a potentially life-threatening condition that emerges when the host response to infections exceeds normal limits and impairs vital organ functions (1–3). Sepsis is still one of the leading causes of death in the world, as a heavy burden and colossal menace to the critical patients (3, 4). According to literature reports, more than 750,000 individuals suffered from sepsis and 200,000 deaths are due to sepsis per year in the USA (5, 6). Despite the great advancements made in the recognition and treatment of sepsis, the mortality in hospitalized sepsis patients remains very high at 25–30% (4, 7–9).

In 2016, the Third International Consensus Definition for Sepsis and Septic Shock (Sepsis 3.0) revised the definition of sepsis as a life-threatening organ dysfunction caused by dysregulated host responses to infection (10). Sepsis 3.0 reflects the nature of the disease based on a better understanding of sepsis. However, the precise mechanisms of cell injuries and systemic organ dysfunction induced by sepsis are still not elucidated clearly. As is known, the immune system plays a critical role throughout the course of sepsis, which is characterized by systemic dysregulated immune response to infection and leads to microvasculature and endothelial impairment (4, 11). The normal regulation of the immune system is conducive to clear the source of infection and limit the intensity of the inflammatory response.

Thus, immune-related biomarkers, such as neutrophil-to-lymphocyte ratio (NLR), C-reactive protein (CRP), procalcitonin (PCT), sTREM-1, presepsin, cytokines, and various interleukins, are increasingly being evaluated for their abilities of early recognition and to predict prognosis of sepsis (5, 12–14). NLR has been found to serve as a convenient prognostic marker in septic patients despite numerous pieces of evidence that reported the correlation between NLR and adverse outcomes of sepsis remained controversial (12, 15). As a sign of inflammation, NLR is derived from the absolute counts of neutrophils and absolute lymphocytes. Lymphopenia due to lymphocyte apoptosis is a prominent feature of sepsis, which may account for adaptive immunodepression (16). Neutrophils play a critical role in protecting the body from infections by a variety of pathogenic microorganisms. Therefore, a dramatical increase in neutrophil count has been found in severe infectious diseases, especially in sepsis, which reflects the severity of the infection. The underlying mechanism may include: 1) The increased lifespan during sepsis due to the over-expression of anti-apoptotic protein Mcl (myeloid cell leukemia)-1 (17) and resistance to apoptosis contributed by several signal pathways in neutrophils, including extracellular regulated protein kinases (ERKs) 1/2, phosphoinositide-3 kinases (PI-3K)/Akt, Src-homology domain 2 containing tyrosine phosphatase-1 (SHP-1)/caspase-8, *etc.* (18); 2) Increased production and release of both mature and immature neutrophils driven by the up-regulated level of granulocyte colony-stimulating factor (G-CSF) and the unbalance of chemokines and their receptors (19). Hence, NLR is a valuable biomarker significantly associated with the immune status. Whereas, NLR only reflects the change in the quantity of immune cells instead of functional change. As a crucial member of inflammatory cytokines, interleukin-6 (IL-6) can be produced by multiple immune cells and plays a pivotal role in host responses to infections by regulating immune function (20–22). Accordingly, combining NLR with IL-6 might potentially enhance the ability to predict the death risk of patients with sepsis. In this study, we aimed to evaluate the effects of NLR combined with IL-6 on admission day in predicting 28-day mortality of septic patients.

## Material and Methods

### Participant Enrollment and Study Design

**Figure 1:**
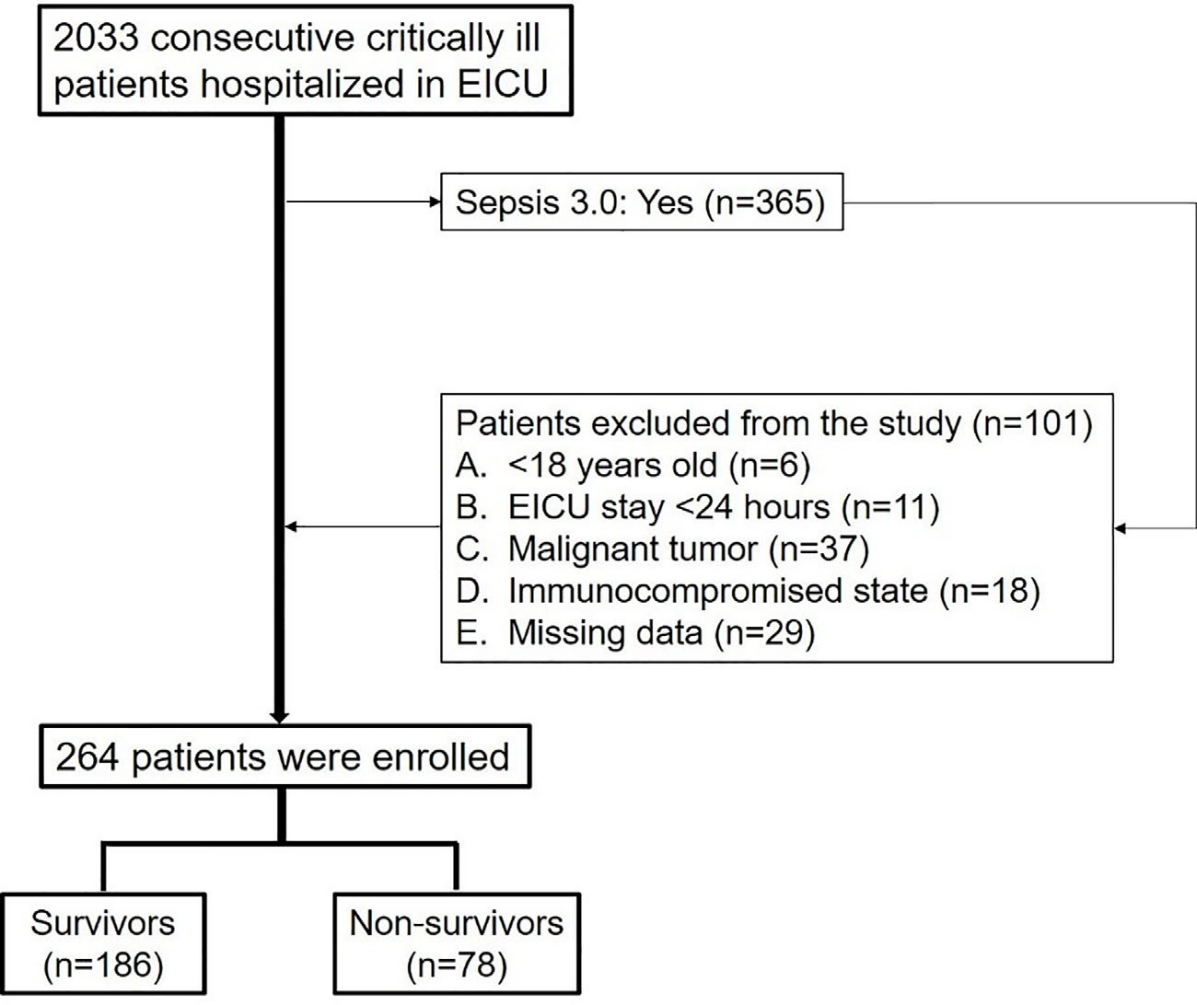
Flowchart of the enrolled patients.

This is a single-center retrospective observational study performed at a tertiary teaching hospital. The study period was from May 1^st^, 2017 to April 30^th^, 2020, and 2,033 critically ill patients were admitted to the Emergency Intensive Care Unit (EICU) of the Fourth Medical Center of the Chinese PLA General Hospital, Beijing, China (**Figure 1**). Adult patients older than 18 years old who met the diagnostic criteria of sepsis 3.0 (10) were enrolled. The exclusion criteria were including younger than 18 years old, missing data, EICU stay <24 h, malignant tumor, or immunocompromised state (*eg*, HIV positive or long-term use of glucocorticoids, immunosuppressants). All the patients selected in the study were observed for at least 28 days.

The patients received 24-h medical monitoring and care provided by the staff and clinicians in our EICU. The therapeutic strategy was guided following the instructions of Surviving Sepsis Campaign Guideline (10). This study complied with the Declaration of Helsinki and was approved by the ethics committee of the Fourth Medical Center of the Chinese PLA General Hospital, Beijing, China. Throughout the study period, all the data were obtained routinely (the IL-6 test is a routine test item in our hospital) for the diagnosis or treatment of sepsis, for which the informed consent was not required by the institutional review board.

### Data Collection

We collected the patient data from the electronic and paper medical records and recorded them on a designed record form, including demographic and clinical data: (1) demographics: age, sex, and body mass index (BMI); (2) vital signs on admission: body temperature, systolic blood pressure (SBP), and heart rate (HR); (3) source of infection; (4) underlying diseases; (5) laboratory results: blood routine tests, IL-6, PCT, and CRP tests; (6) Severity scores: Sequential Organ Failure Assessment (SOFA) scores and Acute Physiology and Chronic Health Evaluation (APACHE) II scores.

### Laboratory Parameter Examination

Blood samples for the blood routine tests and other laboratory parameter examination were obtained from venous puncture after presentation to the EICU and were analyzed within 3 h. The routine blood tests were performed by using an XN-10 (B4) automated hematology analyzer (Sysmex, Kobe, Japan). The PCT and IL-6 were measured using the electrochemical luminescence immunoassay system (Roche, cobas e411), and the CRP was examined by using the CRP quantitative analyzer (QuickRead go system).

### Statistical Analysis

The measurement data were expressed as mean ± standard deviation (SD), percentage or median (interquartile range), which were divided into two categories: continuous and categorical variables. Comparison of two groups was performed using chi-square test for categorical variables, as well as student *t* test or Mann–Whitney *U* tests for continuous variables. The box plots were performed to examine the correlation between serum levels of biomarkers (CRP, PCT, NLR and IL-6) and severity scores (defined as APACHE II and SOFA scores). To evaluate whether the above biomarkers have potential predictive value of 28-day mortality in sepsis, we employed a Cox regression model. In the univariate analysis, the data were compared by using odds ratios within 95% confidence intervals (CIs). Multivariate Cox regression models were used to confirm the relationship between the studied parameters and 28-day mortality in model 1 (adjustment for APACHE II scores and SOFA scores) and model 2 (adjustment for age, sex, BMI, SBP, APACHE II and SOFA scores).

The predictive accuracy of each model was evaluated by using three different statistical methods. Firstly, the area under the receiver-operating characteristic (ROC) curve (AUC), for which larger values suggest better efficacy of discrimination was evaluated. We compared the AUC value among different biomarkers using the method of DeLong et al. (23). Next, we assessed the ability of the studied biomarkers (CRP, PCT, NLR, and IL-6) to accurately classify patients with sepsis into more appropriate mortality risk categories by using the net reclassification improvement (NRI) and the integrated discrimination improvement (IDI), which are commonly used measures of risk prediction model performance (24, 25). The IDI index reflects the improvement in both discrimination and reclassification, and the NRI index is used to evaluate the improvement of the new model in the prediction probability (25).

In addition, the best clinical cut-off value, sensitivity, specificity, Youden index, positive predictive value (PPV), and negative predictive value (NPV) for each parameter were calculated in predicting the 28-day mortality in septic patients. Last, the survival was estimated by the Kaplan–Meier method, and the differences in survival were compared with a log-rank test. All statistical analyses were performed with SPSS^®^ (version 22.0, Chicago, USA) and MedCalc^®^ (version 11.4.40, Belgium). In addition, the NRI and IDI statistics were computed by using R Statistical Software (version 4.0.3, Vienna, Austria). A two-sided *P* value <0.05 was considered statistically significant.

## Results

### Patient Demographics and Clinical Data

During the study period, a total of 264 patients with sepsis who meet the criteria were enrolled in this study. The baseline characteristics of these patients are shown in **Table 1**. In all, 186 (70.5%) patients survived more than 28 days. There were no significant differences in sex, age, and heart rates between the survivors and non-survivors (*P* > 0.05). The non-survivors were characterized as having higher BMIs, higher body temperatures, and lower SBPs, as well as a higher rate of lower respiratory tract infections (50%) (*P* < 0.05) in the present study. In general, non-survivors had much higher disease severity scores, worse blood testing results including NLR, IL-6, and PCT, and had heavier burden of comorbidities and organ dysfunction (*P* < 0.05). Moreover, the lactate levels in the non-survivors were significantly higher than in the survivors (**Supplementary Table S1**, *P* < 0.01). A total of 38 patients (48.7%) progressed to septic shock in the non-survivors, and fewer patients (11.8%) underwent septic shock in the survivors (*P* < 0.01). Similarly, much higher rate of bacteremia, vasopressors using, intubation, and glucocorticosteroid using was found in the non-survivors (**Supplementary Table S1**, *P* < 0.01).

**Table 1:** Baseline characteristics of the 264 patients with sepsis.

Variables	Total n=264	Survivor n=186	Non-survivor n=78	*P* value
**Demographics and vital signs**				
Sex (male %)	167 (63.3)	113 (60.8)	54 (69.2)	0.192
Age, years	52.94 ± 12.61	52.05 ± 12.97	55.06 ± 11.51	0.077
BMI, kg/m^2^	21.98 ± 2.46	21.69 ± 2.12	22.67 ± 3.04	0.011
Body temperature, °C	37.96 ± 0.87	37.85 ± 0.89	38.24 ± 0.76	0.001
SBP, mmHg	113.79 ± 18.36	118.55 ± 18.42	102.42 ± 12.29	0.001
Heart rate, bpm	98.06 ± 17.25	97.65 ± 17.99	99.04 ± 15.41	0.550
**Site of primary infection**				
Lower respiratory tract	105 (39.8)	66 (35.5)	39 (50.0)	0.006
Intra-abdomen	58 (22.0)	48 (25.8)	10 (12.8)	
Urinary system	40 (15.2)	33 (17.7)	7 (9.0)	
Skin and soft tissue	52 (19.7)	31 (16.7)	21 (26.9)	
Unknown origin	9 (3.4)	8 (4.3)	1 (1.3)	
**Comorbidities**				
Hypertension	71 (26.9)	41 (22.0)	30 (38.5)	0.006
Diabetes mellitus	47 (17.8)	28 (15.1)	19 (24.4)	0.071
CHD	37 (14.0)	17 (9.1)	20 (25.6)	0.001
COPD	19 (7.2)	11 (5.9)	8 (10.3)	0.213
**Laboratory data**				
NLR	4.80 ± 1.94	4.19 ± 1.43	6.24 ± 2.23	0.001
IL-6, pg/ml	87.07 ± 10.99	75.88 ± 10.04	113.77 ± 11.69	0.001
PCT, ng/ml	6.73 ± 4.83	5.36 ± 3.57	10.01 ± 5.79	0.001
CRP, mg/L	89.97 ± 2.68	87.98 ± 2.58	94.72 ± 3.75	0.058
**Severity scores**				
APACHE II	20.79 ± 6.89	17.65 ± 4.01	28.29 ± 6.52	0.001
SOFA	8 (6–11)	7 (5–9)	12 (8–16)	0.001

Data are expressed as mean ± SD, median (interquartile range), or No. (%). P-value <0.05 indicates statistical significance.

CHD, coronary heart disease; COPD, chronic obstructive pulmonary disease.

### Comparison of Correlation of Studied Biomarkers and Severity Scores

**Figure 2:**
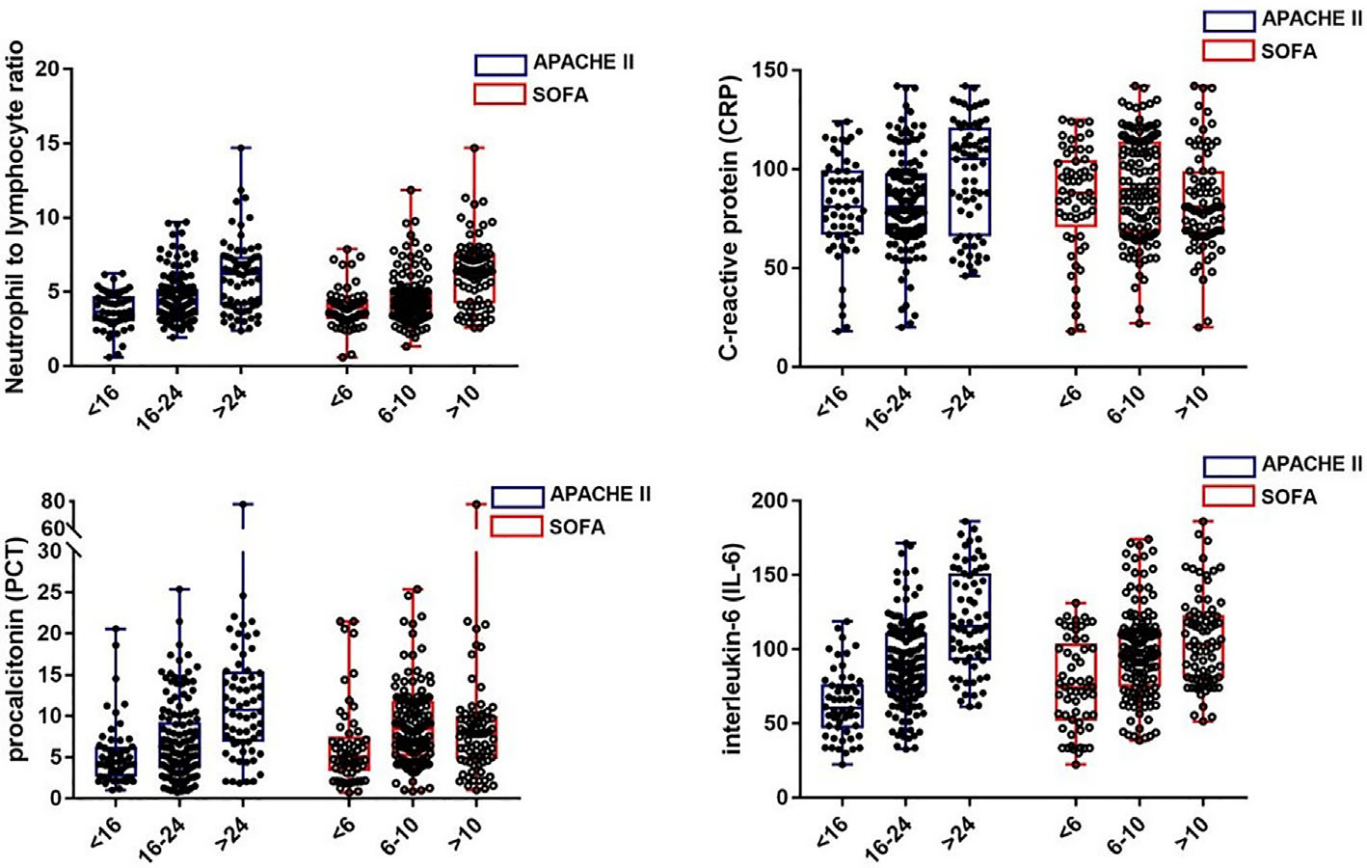
The levels of NLR, CRP, PCT, and IL-6 in septic patients with different severity classifications.

We examined the correlation between four laboratory markers (NLR, IL-6, PCT, and CRP) and the severity scores (APACHE II and SOFA scores). According to the previous studies, we separated the patients into three groups according to the illness scoring systems: (1) APACHE II scores: <16, 16–24,>24; and (2) SOFA scores: <6, 6–10, >10 (26, 27). The scatter plots of the four biomarkers stratified by APACHE II and SOFA scores are displayed in **Figure 2**. It was found that IL-6 had the strongest correlation with both APACHE II and SOFA scores, followed by the NLR and PCT, and there was no obvious correlation between CRP and the illness severity scores.

### Predictors of the 28-Day Mortality in Septic Patients

As revealed in **Table 2**, in the univariate Cox proportional hazards model, NLR, IL-6, and PCT were associated with 28-day mortality of septic patients (*P* < 0.001). In the multivariable Cox proportional hazards model 1 and model 2, NLR and IL-6 were found to be associated with 28-day mortality (*P* < 0.01). After adjusting for age, sex, BMI, SBP, APACHE II and SOFA scores, NLR and IL-6 were considered as independent predictors of the 28-day mortality in septic patients.

**Table 2:** Univariate and multivariate Cox regression models to predict 28-day mortality.

Variables	Univariate		Multivariate (model 1)		Multivariate (model 2)	
	Odds ratio (95%CI)	*P*	Odds ratio (95%CI)	*P*	Odds ratio (95%CI)	*P*
NLR	1.340(1.253–1.434)	<0.001	1.283(1.167–1.410)	<0.001	1.281(1.159–1.414)	<0.001
IL-6	1.029(1.023–1.035)	<0.001	1.015(1.004–1.026)	0.008	1.017(1.005–1.028)	0.004
PCT	1.050(1.037–1.064)	<0.001	0.999(0.978–1.020)	0.920	0.994(0.970–1.019)	0.656
CRP	1.009(1.000–1.018)	0.051	0.998(0.985–1.010)	0.703	0.996(0.983–1.009)	0.555

Model 1 included APACHE II and SOFA scores. Model 2 included age, sex, BMI, SBP, APACHE II, and SOFA scores. P-value < 0.05 indicates statistical significance.

NLR, neutrophil to lymphocyte ratio; CRP, C-reactive protein; PCT, procalcitonin; IL-6, interleukin-6.

### The Predictive Efficacy of 28-Day Mortality for Studied Parameters

**Figure 3:**
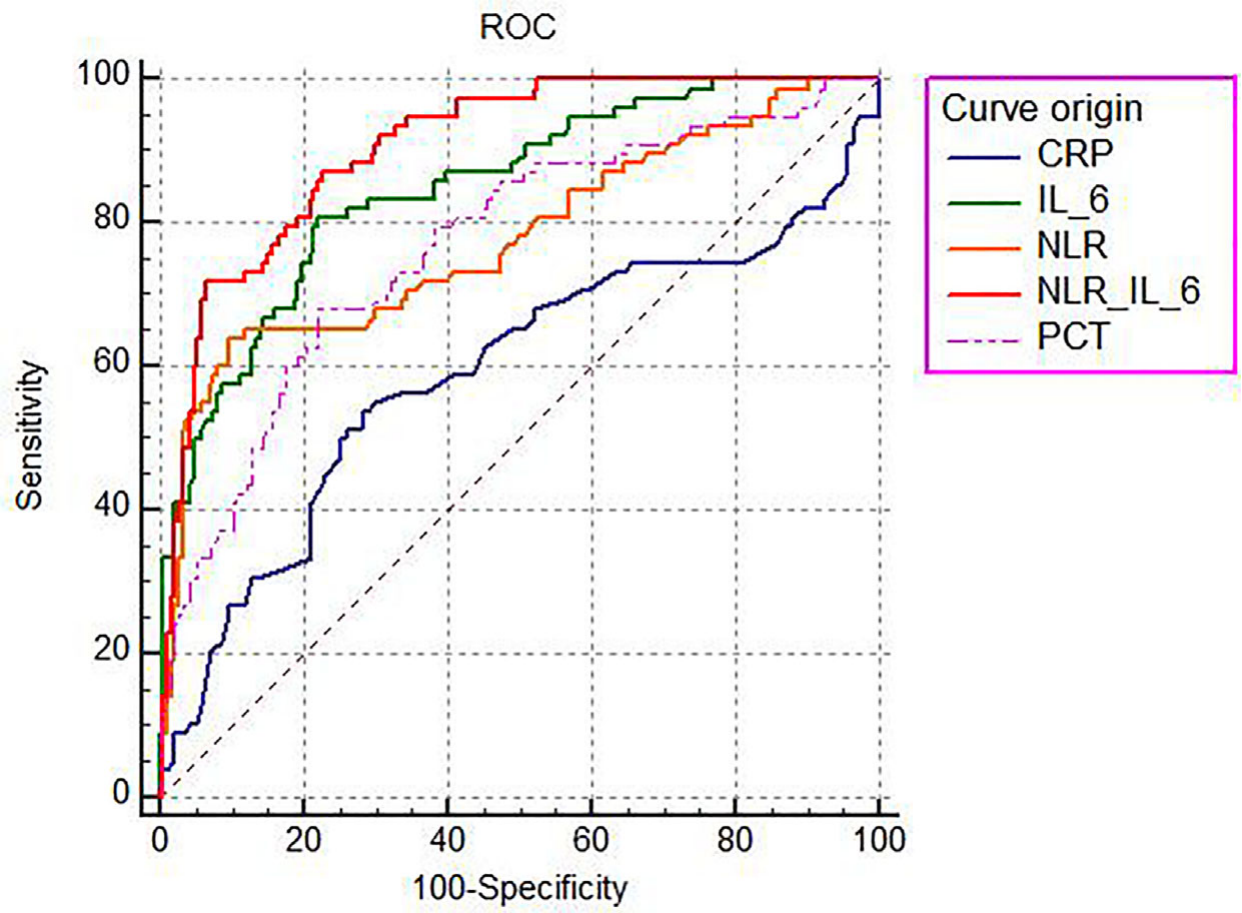
The ROC analysis of the studied biomarkers for predicting the development of 28-day mortality in sepsis.

The ability of the studied parameters to discriminate the 28-day mortality was defined as the area under the curve (AUC) and was evaluated by using the receiver operating characteristic curve (ROC) analysis (**Figure 3**). The AUC values of CRP, PCT, NLR, IL-6, and NLR plus IL-6 (NLR_IL-6) were 0.591 (0.529–0.651), 0.768 (0.713–0.818), 0.776 (0.721–0.825), 0.849 (0.799–0.890), and 0.904 (0.862–0.937), respectively. We compared AUC value among the different biomarkers and found that NLR_IL-6 presented the largest AUC value than the other variables (**Table 3**, *P* < 0.01).

**Table 3:** The discriminating capability of different biomarkers in predicting 28-day mortality of septic patients.

Variables	CRP	PCT	NLR	IL-6	NLR_IL-6
AUC	0.591	0.768	0.776	0.849	0.904
	(0.529–0.651)	(0.713–0.818)	(0.721–0.825)	(0.799–0.890)	(0.862–0.937)
	NA	*P* = 0.0001	*P* = 0.8658	*P* = 0.1040	*P* = 0.0028
IDI	NA	0.266	0.074	0.167	0.197
		−0.062–0.476	−0.016–0.171	0.024–0.338	0.030–0.248
		*P* < 0.001	*P* = 0.109	*P* = 0.03	*P* = 0.020
NRI	NA	0.074	0.263	0.363	0.246
		0.008–0.108	0.011–0.447	0.128–0.561	−0.071–0.473
		*P* = 0.239	*P* = 0.040	*P* < 0.001	*P* = 0.019

Pairwise statistical comparisons are performed from left to right. P-value less than 0.05 is considered statistically significant.

AUC, Area under the ROC curve; IDI, Integrated discrimination improvement; NRI, Net reclassification improvement.

Next, we further compared the reclassification improvement of the studied biomarkers in the reclassification ability of 28-day mortality risk in sepsis patients (**Table 3**). When comparing PCT to CRP, it was noted that PCT showed a higher IDI than CRP; however, it failed to demonstrate a higher NRI than CRP. In addition, NLR presented a higher NRI alone compared with PCT. Moreover, IL-6 could better reclassify patients into a more appropriate 28-day mortality risk category than CRP, PCT, and NLR, as indicated by significantly higher IDI and NRI. Notably, our data reflected that distinctly better IDI and NRI than IL-6 appeared when we combined NLR and IL-6.

As shown in **Table 4**, we calculated the sensitivity, specificity, cut-off point, PPV, NPV, and Youden index to further assess the predictive value of each laboratory marker comprehensively. We found that IL-6 had the highest sensitivity (80.77%, 70.3–88.8%), and NLR_IL-6 had the highest specificity (93.55%, 89.0–96.6%) with a relatively high sensitivity (71.79%, 60.5–81.4%) for 28-day mortality prediction. The cut-off point of each biomarker was listed in **Table 4**. Based on the cut-off values, our date showed that NLR_IL-6 had the best PPV (82.4%, 72.6–89.1%), and IL-6 had the best NPV (90.6%, 85.9–93.9%). Furthermore, NLR_IL-6 had the highest Youden index (0.5873). Collectively, NLR_IL-6 performed best in prediction, discrimination, and reclassification of the 28-day mortality risk in sepsis.

**Table 4:** Comparison of the sensitivity and specificity of the studied biomarkers in predicting 28-day mortality.

Variables	Sensitivity (95% CI)	Specificity (%)	Cut-off point	PPV (95% CI)	NPV (95% CI)	Youden index
CRP	53.85	71.51	102	44.2	78.7%	0.2535
	42.2–65.2	64.4–77.9		36.8–51.9	74.1–82.7	
PCT	67.95%	77.96%	6.54	56.4%	85.3%	0.4591
	56.4–78.1	71.3–83.7		48.7–63.8	80.6–89.0	
NLR	64.10%	90.32%	5.55	73.5%	85.7%	0.5443
	52.4–74.7	85.1–94.2		63.5–81.6	81.6–89.0	
IL-6	80.77%	77.96%	100	60.6%	90.6%	0.5873
	70.3–88.8	71.3–83.7		53.5–67.3	85.9–93.9	
NLR_IL-6	71.79%	93.55%	NLR = 4.937 IL-6 = 117.6	82.4%	88.8%	0.6534
	60.5–81.4	89.0–96.6		72.6–89.1	84.7–91.9	

**Figure 4:**
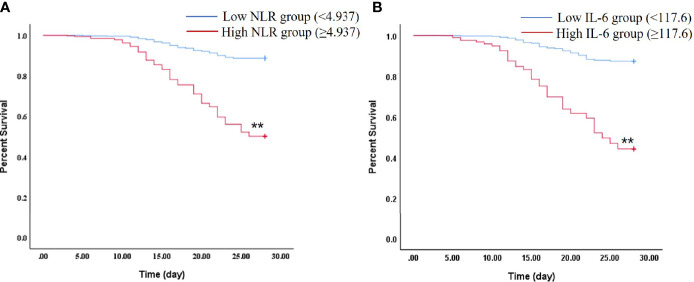
Kaplan–Meier plot showing survival in septic patients grouped by NLR or IL-6 levels. **(A)** Kaplan–Meier survival curves of 28-day mortality according to NLR levels (log-rank = 0.000). ^**^ *P* < 0.01 *vs* Low NLR group. **(B)** Kaplan–Meier survival curves of 28-day mortality according to IL-6 levels (log-rank = 0.000). ^**^ *P* < 0.01 *vs* Low IL-6 group.

We further compared the 28-day mortality risk in septic patients according to the cut-off point of NLR and IL-6. As shown in the **Supplementary Table S2**, 41 of 64 patients with high NLR (≥4.937) died, and 163 of 200 patients with low NLR (<4.937) survived (*P* < 0.01). In addition, it was also noticed that septic patients in the high IL-6 (≥117.6) group had a significantly higher 28-day mortality than the low IL-6 (<117.6) group (*P* < 0.01). Kaplan–Meier survival curves were performed and compared between the septic patients stratified by the NLR or IL-6 cut-off points. It was found that patients with NLR or IL-6 levels below the cut-off point had significantly higher survival rate (**Figure 4**, *P* < 0.01).

## Discussion

Sepsis is caused by dysregulated immune responses to the systemic infections, which leads to multiple organ dysfunction and is a potentially lethal complication in the ICU (1–3, 28). Thus, early identification of the risk of death from sepsis is important for the timely and effective management and intervention. The status of immune functions and the intensity of the inflammatory response impact the outcomes of patients with sepsis profoundly (29–31). Therefore, we noticed that more patients suffered from hemodynamic instability and adrenal insufficiency in the non-survivors due to immune paralysis and aggressive inflammatory response. NLR and IL-6 are both clinically accessible biomarkers which are closely related to the occurrence and development of sepsis. It is of great value for the treatment of sepsis to explore the deep meaning and hidden important clinical information of NLR plus IL-6. Accordingly, the present study sought to develop a new death risk screening tool defined as NLR combined with IL-6, and the 28-day mortality predictive values of several biomarkers in sepsis were evaluated and compared.

As we know, neutrophils play a vital role in innate immunity, as the first line of defense cells providing rapid sensing and elimination of pathogens. Moreover, lymphocytes are the key cell type of adaptive immunity, which provide a broader and more finely tuned repertoire of recognition for the antigens (32, 33). Therefore, the NLR reflects the balance between innate and adaptive immunity. In addition, the NLR is a convenient accessible biomarker that can be analyzed based on the whole blood cell count and has been reported to be associated with various diseases including inflammation, cerebral infarction, cancer, and trauma (12, 33–38). IL-6 is a pleiotropic cytokine and inflammatory biomarker produced in host response to infections and was evaluated for the validity, as a marker, in the diagnosis of sepsis in many studies (39–41).

In our retrospective study, we demonstrated that the NLR, IL-6, and PCT values were significantly higher in the non-survivors than in the survivors (*P* < 0.01), which was consistent with the previous studies (15, 40–42). However, there was no significant difference in CRP levels between survivors and non-survivors. These data indicated that the NLR, IL-6, and PCT may serve as potential prognostic markers in sepsis. We further assessed the association between the biomarkers and disease severity. It was noted that IL-6 showed the strongest correlation with the severity of illness, followed by the NLR and PCT, which indicated that these biomarkers might be effective indicators in stratifying disease severity in order to give the prediction the prognosis of the patients with sepsis. Nevertheless, the CRP, as an important acute phase protein, showed no significant correlation with the disease severity in the current study. Similarly, Mierzchala-Pasierb M et al. (43) conducted an observational and prospective study and reported that there were no significant differences for the CRP levels between any subgroups according to disease seriousness. The possible explanation is that the CRP peaks only at 24–48 h after the onset of infections or tissue damage (33, 44), which determines the poor timeliness of CRP.

Next, we investigated the factors that independently predicted the 28-day mortality in sepsis, which is of high importance for the appropriate and effective interventions. Our data showed that the NLR and IL-6 were independent predictors of the 28-day mortality in septic patients after adjustment for age, sex, BMI, SBP, APACHE II, and SOFA scores, by using Cox multivariate models. These results were consistent with those reported by several other studies (12, 15, 41, 45). Furthermore, the ROC curves were used to evaluate the predictive power of each of the above independent factors for the 28-day mortality in sepsis. It was noticed that IL-6 had the largest AUC value (0.849), followed by NLR (0.776), PCT (0.768), and CRP (0.591) as a single parameter. Notably, a significant larger AUC (0.904) was found after we combined the NLR with IL-6 as a composite index (*P* < 0.01). In addition, the optimum cut-off values for the studied parameters were identified by drawing ROC curves. And, the cut-off of the IL-6 was found to be 100 pg/ml; the sensitivity, specificity, PPV, and NPV were 80.77, 77.96, 60.6, and 90.6%, respectively. The cut-off point of NLR was 5.55, with the sensitivity of 64.10%, specificity of 90.32, PPV of 73.5%, and NPV of 85.7%, which was in line with the previous studies (46, 47). Importantly, IL-6 combined with NLR could markedly enhance the specificity (93.55%) and PPV (82.4%), in which the cut-off values were 4.937 for NLR and 117.6 pg/ml for IL-6. And, Kaplan–Meier curve analysis indicated that septic patients with NLR and IL-6 levels above the cut-off points had higher 28-day mortality risk than those with low NLR and IL-6 levels.

Moreover, we used the IDI and NRI to further measure the death risk prediction of the studied biomarkers, by which the overall reclassification improvement of these biomarkers was examined (25, 48). In the original work, NLR_IL-6 had an IDI of 0.197, with comparison of IL-6, indicating that the NLR_IL-6 model could significantly improve the mean difference of predicted probabilities for survival and death by 19.7% (*P* < 0.05). Additionally, we calculated the NRI to evaluate the true discriminatory potential of different biomarkers and their combination. The NRI reflects the incremental strength of a new prediction model compared with the old prediction model, which can be divided into three levels: >0.6 (strong), 0.2–0.6 (medium), and <0.2 (weak) (25). In the current study, the NRI of NLR_IL-6 is 0.246 when compared with IL-6 (*P* = 0.019), suggesting that NLR_IL-6 could significantly improve the ability to predict the risk of death of septic patients than other biomarkers.

Our study has some strengths. First, the sample size of our study is relatively large in order to reduce selection bias. Second, to the best of our knowledge, our work firstly demonstrated the enhanced prognostic prediction value of NLR combined IL-6, which could guide the clinicians to adopt more appropriate and accurate management for sepsis patients. Third, we performed several probability models to evaluate the prediction power of the studied biomarkers by rigorous statistical analyses, in order to ensure the scientific nature and credibility of the results.

Nevertheless, this study has several limitations. First, a key limitation is that our work is a single institutional retrospective study. A prospective multicenter study is needed to further confirm our conclusions. Second, we could not conclude that a higher NLR or IL-6 level represents a fatal immune disorder or inflammatory storm from our study. Furthermore, the pathophysiological basis leading to the death of patients with sepsis could not be elucidated in this study. Finally, we did not mention much about the role of hemodynamic effects in the mortality risk of septic patients. Septic shock is characterized by uncontrolled host response to infections, as the severe form and main cause of death of sepsis (49). Thus, the hemodynamic status maybe of higher value in predicting the risk of death in septic patients, which is the main subject of our forthcoming study.

## Conclusion

In summary, the present study demonstrated that the levels of NLR and IL-6 are associated with the severity of disease in septic patients and appear to be independent predictors of 28-day mortality of septic patients. Importantly, NLR combined IL-6 could markedly enhance the prediction power of 28-day mortality of patients with sepsis than the single biomarker.

## Data Availability Statement

The raw data supporting the conclusions of this article will be made available by the authors, without undue reservation.

## Ethics Statement

The studies involving human participants were reviewed and approved by the ethics committee of the Fourth Medical Center of the Chinese PLA General Hospital. The patients/participants provided their written informed consent to participate in this study.

## Author Contributions

These authors have contributed equally to this work and share first authorship. All authors contributed to the article and approved the submitted version.

## Conflict of Interest

The authors declare that the research was conducted in the absence of any commercial or financial relationships that could be construed as a potential conflict of interest.

The reviewer LX declared a shared affiliation with the authors to the handling editor at the time of review.
