# Supplementary material for: Effects of Neutrophil-to-Lymphocyte Ratio Combined With Interleukin-6 in Predicting 28-Day Mortality in Patients With Sepsis
Source: Front Immunol. 2021 Mar 16;12:639735. doi: 10.3389/fimmu.2021.639735 (PMC8007868; doi:10.3389/fimmu.2021.639735)
Supplement: Supplementary file 1 [file Table_1.docx]

Supplementary Table S1. Comparison of lactate level, vasopressors, septic shock, bacteremia, intubation and mechanical ventilation, and glucocorticosteroid use between the two groups.

| Parameters | Total | Survivors | Non-survivors | *P* value |
| --- | --- | --- | --- | --- |
|  | (n=264) | (n=186) | (n=78) |  |
| Lactate (mmol/L) | 1.78±0.64 | 1.72±0.58 | 1.94±0.76 | 0.009 |
| Septic shock (n, %) | 60 (22.7) | 22 (11.8) | 38 (48.7) | <0.01 |
| Vasopressors (n, %) | 79 (29.9) | 33 (17.7) | 46 (59.0) | <0.01 |
| Bacteremia (n, %) | 125 (47.3) | 74 (39.8) | 51 (65.4) | <0.01 |
| Intubation (n, %) | 73 (27.7) | 42 (22.6) | 31 (39.7) | 0.004 |
| Mechanical ventilation duration (hour) | 194  (29-562) | 159.5  (29-562) | 259  (108-511) | 0.001 |
| Glucocorticosteroid use (n, %) | 43 (16.3) | 14 (7.5) | 29 (37.2) | <0.01 |

Data are presented as mean ± SD, number (%), or median (interquartile range).

Supplementary Table S2. Distributions of septic patients in the survivors and non-survivors according to NLR and IL-6 level status.

| Groups | Survivors | Non-survivors | *P* value |
| --- | --- | --- | --- |
|  | (n=186) | (n=78) |  |
| High NLR (≥4.937) | 23 | 41 | <0.01 |
| Low NLR (<4.937) | 163 | 37 |  |
| High IL-6 (≥117.6) | 15 | 35 | <0.01 |
| Low IL-6 (<117.6) | 171 | 43 |  |
